# Calcitonin gene-related peptide antagonists in pregnancy: a disproportionality analysis in VigiBase^®^

**DOI:** 10.1186/s10194-024-01715-4

**Published:** 2024-01-19

**Authors:** Roberta Noseda, Francesca Bedussi, Claudio Gobbi, Alessandro Ceschi, Chiara Zecca

**Affiliations:** 1 Division of Clinical Pharmacology and Toxicology, Institute of Pharmacological Sciences of Southern Switzerland, Ente Ospedaliero Cantonale, Lugano, Switzerland; 2 Department of Neurology, Neurocenter of Southern Switzerland, Ente Ospedaliero Cantonale, Lugano, Switzerland; 3 Faculty of Biomedical Sciences, Università della Svizzera Italiana, Lugano, Switzerland; 4 Department of Neurology, University Hospital Basel, Basel, Switzerland; 5 Clinical Trial Unit, Ente Ospedaliero Cantonale, Lugano, Switzerland; 6 Department of Clinical Pharmacology and Toxicology, University Hospital Zurich, Zurich, Switzerland

**Keywords:** Calcitonin gene–related peptide antagonists, Safety, Pregnancy, Disproportionality, VigiBase

## Abstract

**Background:**

Current evidence on the safety of calcitonin gene–related peptide antagonists (CGRP-A) in pregnancy for the treatment of both episodic and chronic migraine is scarce and does not yet provide definitive information. By querying VigiBase^®^, the World Health Organization global pharmacovigilance database, this study aimed to detect differences in the reporting frequency between CGRP-A and triptans in relation to pregnancy.

**Methods:**

Disproportionality analyses on de-duplicated safety reports collected in VigiBase^®^ as of 31.05.2023 reporting exposure to CGRP-A in pregnancy with or without pregnancy outcomes. A Reporting Odds Ratio (ROR) with a 95% confidence interval (CI) was used as a measure of disproportionality and the threshold for the detection of a signal of disproportionate reporting was set with a 95% CI lower limit > 1.

**Findings:**

Four hundred sixty-seven safety reports reported exposure to CGRP-A in pregnancy, mostly originating from the United States of America (360/467, 77%), more frequently reported by patients (225/467, 48%), who were mainly females (431/467, 92%), and more frequently reported exposure to CGRP-A during pregnancy (400/467, 86%). Compared to triptans, no signals of disproportionate reporting were detected with CGRP-A either for the overall reporting of pregnancy-related safety reports (ROR 0.91, 95% CI 0.78–1.06), for the reporting of pregnancy outcomes (maternal and/or foetal/neonatal, ROR 0.54, 95% CI 0.45–0.66), or for the reporting of foetal/neonatal outcomes (ROR 0.53, 95% CI 0.41–0.68).

**Conclusions:**

This study showed that, to date, there are no signals of increased reporting with CGRP-A compared to triptans in relation to pregnancy in VigiBase^®^. Future pharmacovigilance studies are needed to confirm these findings.

**Supplementary Information:**

The online version contains supplementary material available at 10.1186/s10194-024-01715-4.

## Introduction

The relationship between migraine and pregnancy is highly variable [1, 2], with the majority of women experiencing an improvement in their symptoms during pregnancy (55 to 90% of cases) [3], while others reporting an unchanged, or rarely, a worsened migraine course during pregnancy especially in the first trimester (8% of cases) [4].

Since 2018, two drug classes inhibiting the signalling of calcitonin gene-related peptide (CGRP) have become available as acute or preventive migraine treatments [5, 6]: monoclonal antibodies targeting CGRP or its receptor and the small-molecule CGRP receptor antagonists (gepants), collectively referred to as CGRP antagonists (CGRP-A).

CGRP-A are effective in both episodic and chronic migraine according to randomized controlled trials and several observational studies [7]. However, safety data on their use in pregnancy are limited to anti-CGRP monoclonal antibodies and stem predominantly from single clinical cases [8–10] and pharmacovigilance studies on spontaneous safety reports [11]. Conversely, no safety data in humans on the use of gepants in pregnancy are currently available.

Regardless of the two different therapeutic modalities by which CGRP-A act and the different timing of crossing the placental barrier, it seems reasonable to expect similar consequences from their use in human pregnancy when considering the role of CGRP in the development and regulation of the utero-placental blood flow [12].

To gain further information on the safety of CGRP-A when used in pregnancy, we queried VigiBase^®^, the World Health Organization global database of spontaneous safety reports, and performed disproportionality analyses to detect differences in the reporting frequency between CGRP-A and the migraine-specific acute treatment with triptans [13] in relation to pregnancy.

## Methods

Disproportionality analyses were performed on de-duplicated spontaneous safety reports collected in VigiBase^®^ as of 31.05.2023. Drugs of interest, selected as active ingredients, included suspected monoclonal antibodies erenumab (targeting CGRP receptor), galcanezumab, fremanezumab and eptinezumab (targeting CGRP-ligand), and gepants ubrogepant, rimegepant, and atogepant (all targeting CGRP receptor). Events of interest were captured by using the Standardized Medical Dictionary for Regulatory Activities (MedDRA^®^) Query (SMQ) “pregnancy and neonatal topics” (version 26.0). Safety reports reporting as suspected drug(s) sumatriptan, naratriptan, zolmitriptan, rizatriptan, almotriptan, eletriptan and/or frovatriptan were used for the single comparator group (Supplementary Fig. 1), to control for confounding by indication [14], and because, in the absence to date of migraine-specific preventive drugs proven safe in pregnancy, use of triptans in pregnancy appears safe [15]. Safety reports with additional suspected/interacting drugs beyond those of interest and safety reports lacking specific terms referring to drug exposure in pregnancy (including “maternal exposure before pregnancy”, “foetal exposure during pregnancy”, “maternal exposure during pregnancy”, “maternal exposure during breastfeeding”, “paternal exposure during pregnancy”, “maternal exposure time unspecified”) were excluded from the study cohort.

Reporting Odds Ratio (ROR) was used as disproportionality measurement along with its 95% confidence interval (CI) and computed when a minimum number of 5 safety reports of interest was present to reduce the likelihood of false positives [16]. Threshold for the detection of a signal of disproportionate reporting was set with 95% CI lower limit > 1 [16]. The primary outcome was to detect signals of disproportionate reporting for pregnancy exposures to CGRP-A regardless of the reporting of pregnancy outcomes in addition to drug exposure.

The secondary outcomes were i) to detect signals of disproportionate reporting for pregnancy exposures to CGRP-A reporting any pregnancy outcomes (maternal and/or foetal/neonatal); and ii) to detect signals of disproportionate reporting for pregnancy exposures to CGRP-A reporting foetal/neonatal outcomes.

The following sensitivity analyses were performed to control for confounding: i) temporal restriction, starting from 01.01.2018 (when the first in class erenumab received marketing authorization); and ii) temporal restriction and subgroup disproportionality analyses by therapeutic modality (i.e. monoclonal antibodies versus gepants). Data management and analyses were performed with Statistical Analysis System Software (version 9.4; SA Institute, Cary, NC).

According to the Human Research Act (810.30, of 30 September 2011 - status as of 1 September 2023, Art. 2), from the Federal Assembly of the Swiss Confederation, ethical approval and written informed consents were not required.

## Results

### Safety reports’ characteristics

As of 31.05.2023, there were 83′587 de-duplicated safety reports with CGRP-A in VigiBase^®^. Of these, 81′108 (97%) fulfilled the pre-defined inclusion/exclusion criteria, including 467 (0.6%) safety reports reporting exposures to CGRP-A in pregnancy (with or without pregnancy outcomes) (Supplementary Fig. 2). Most of the safety reports related to CGRP-A and pregnancy originated from the United States of America (360/467, 77%), were more frequently reported by patients (225/467, 48%), who were mainly females (431/467, 92%), and more frequently reported exposure to CGRP-A during pregnancy (400/467, 86%) (Table 1).

**Table 1:** Baseline characteristics of the safety reports included in the study

Characteristic	Safety reports with anti-CGRP mAbs *N* = 386	Safety reports with gepants *N* = 76	Safety reports with both an anti-CGRP mAb and a gepant *N* = 5
**Country**			
United States of America	279 (72)	76 (100)	5 (100)
Europe	80 (21)	-	-
South America	8 (2)	-	-
Asia	8 (2)	-	-
Africa	6 (2)	-	-
Australia	5 (1)	-	-
**Reporting year**			
2019	92 (24)	-	-
2020	74 (19)	2 (3)	-
2021	107 (28)	1 (1)	-
2022	76 (20)	26 (34)	1 (20)
2023 (as of 31/05)	37 (9)	47 (62)	4 (80)
**Reporter**			
Physician	129 (33)	6 (8)	-
Other health professional	78 (20)	16 (21)	1 (20)
Pharmacist	10 (3)	-	-
Patient	167 (43)	54 (71)	4 (80)
Not reported	2 (1)	-	-
**Patient sex**			
Female	356 (92)	70 (92)	5 (100)
Male	9 (2)	2 (3)	-
Not reported	21 (6)	4 (5)	-
**Patient age**			
Reported	157 (41)	35 (46)	4 (80)
Median [Q1-Q3], years	33 [28–36]	34 [30–36]	31 [26–36]
Not reported	229 (59)	41 (54)	1 (20)
**Time of drug exposure in pregnancy**			
Before pregnancy	20 (5)	-	-
During pregnancy	341 (88)	55 (72)	4 (80)
Paternal exposure during pregnancy	2 (1)	-	-
During breastfeeding	13 (3)	7 (9)	-
Unknown	10 (3)	14 (19)	1 (20)
**Suspected drug(s)**	185 (48) galcanezumab	61 (80) rimegepant	2 (40) erenumab and rimegepant
	147 (38) erenumab	10 (13) atogepant	
	54 (14) fremanezumab	5 (7) ubrogepant	1 (20) galcanezumab and rimegepant
			1 (20) fremanezumab and rimegepant
			1 (20) eptinezumab and rimegepant
**Indication**			
Migraine	135 (35)	47 (62)	4 (80)
Migraine prophylaxis	18 (5)	5 (7)	1 (20)
Chronic migraine	12 (3)	-	-
Migraine with aura	1 (0)	-	-
Migraine without aura	2 (1)	-	-
Vestibular migraine	2 (1)	-	-
Cluster headache	1 (0)	-	-
Headache	1 (0)	-	-
Not reported	214 (55)	24 (31)	-
**No. of safety reports reporting only drug exposure in pregnancy**	194 (50)	62 (82)	4 (80)
**No. of safety reports reporting drug exposure in pregnancy and foetal/neonatal toxicity (with or without maternal outcomes)**	122 (32) Live-born infants, *n* = 18 Spontaneous abortion, *n* = 72 Abortion induced, *n* = 1 Foetal death, *n* = 1 Stillbirth, *n* = 1 Spina bifida, *n* = 2 Congenital anomaly (not further specified), *n* = 2 Anencephaly, *n* = 1 Anorectal malformation, *n* = 1 Congenital diaphragmatic hernia, *n* = 1 Congenital urinary tract obstruction, *n* = 1 Gastroschisis, *n* = 1 Meningomyelocele, *n* = 1 Trisomy 15, *n* = 1 Wolff-Parkinson white syndrome, *n* = 1 Foetal growth restriction, *n* = 3 Foetal distress syndrome, *n* = 1 Premature baby, *n* = 4 Jaundice, *n* = 2 Bronchiolitis, *n* = 1 Cerebral haemorrhage and epilepsy, *n* = 1 Constipation, *n* = 1 Ear infection, *n* = 1 Haemangioma, *n* = 1 Lethargy, lip swelling, dyspnoea, *n* = 1 Poor feeding infant, *n* = 1	4 (5) Spontaneous abortion, *n* = 2 Abortion induced, *n* = 1 Fallot’s tetralogy, *n* = 1	1 (20) Spontaneous abortion, *n* = 1

Data are n (%)

*CGR*P calcitonin gene– related peptide, *mAb* monoclonal antibody, *Q* quartile

### Disproportionality analyses

**Fig. 1:**
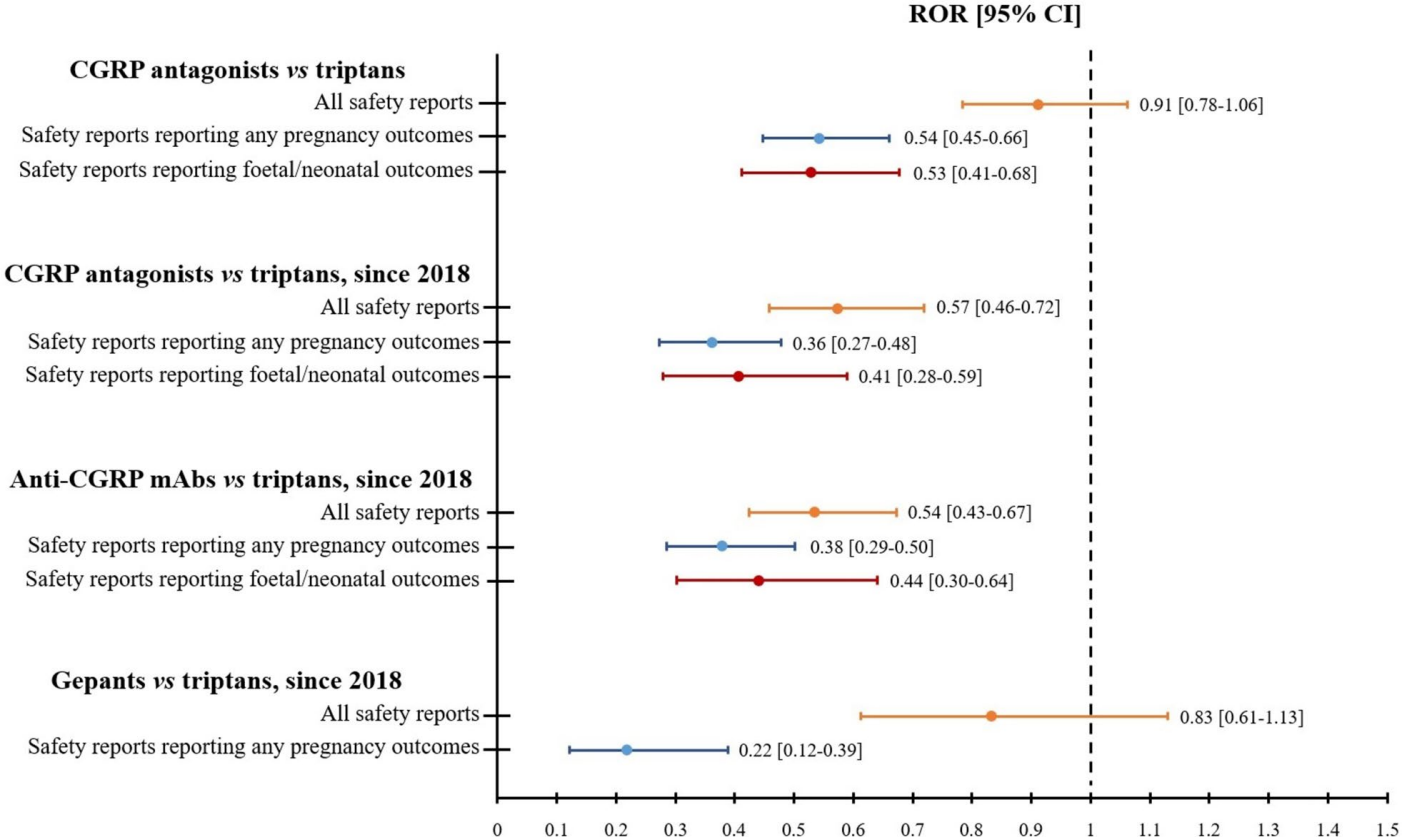
Forest plot representation of the results of disproportionality analyses. *Abbreviations:* CGRP, calcitonin gene-related peptide; ROR, reporting odds ratio; CI, confidence interval; mAbs, monoclonal antibodies

By comparing safety reports associated with CGRP-A against triptans, no signals of disproportionate reporting were detected either for the overall reporting in relation to pregnancy, for the reporting of pregnancy outcomes (maternal and/or foetal/neonatal), or for the reporting of foetal/neonatal outcomes (Fig. 1 and Supplementary Table 1).

Sensitivity analyses assessing the overall reporting of CGRP-A exposure in pregnancy, the reporting of any pregnancy outcomes, and the reporting of foetal/neonatal outcomes since 2018 confirmed these results (Fig. 1 and Supplementary Table 1). Lastly, temporal restriction since 2018 and subgroup disproportionality analyses by therapeutic modality showed that safety reports with anti-CGRP monoclonal antibodies and with gepants, when separately assessed against triptans, were not associated with any signals of increased reporting (overall reporting in relation to pregnancy, reporting of any maternal and/or foetal/neonatal pregnancy outcomes, reporting of foetal/neonatal outcomes) (Fig. 1 and Supplementary Table 1).

## Discussion

We did not detect differences in the reporting frequency between CGRP-A and triptans in relation to pregnancy. This result extended to pregnancy-related safety reports with CGRP-A as a whole (i.e. reporting drug exposure in pregnancy with or without pregnancy outcomes), and reporting pregnancy outcomes, both in general and specifically referring to the foetus/neonate.

To our knowledge, current evidence on CGRP-A safety in human pregnancy does not yet provide definitive information. A handful of single clinical cases [8–10] and a series of 286 safety reports retrieved from VigiBase^®^ by the end of 2021 [11], showed no patterns of maternal, foetal or neonatal toxicity with anti-CGRP monoclonal antibodies, whereas there is still a lack of data on gepants’ safety when used in human pregnancy. Albeit differences between anti-CGRP monoclonal antibodies and gepants in timing of crossing the placental barrier and differences in half-lives [7], we considered CGRP-A as a single group due to the key role played by CGRP in pregnancy, which increases utero-placental blood flow and decreases uterine vascular resistance [12]. Separate disproportionality analyses by therapeutic modality (i.e. monoclonal antibodies versus gepants) confirmed the absence of signals of disproportionate reporting with anti-CGRP monoclonal antibodies in relation to pregnancy, as previously assessed by our group [11]. Also for gepants, no signals of increased reporting in relation to pregnancy were detected. Interestingly, our study also identified five pregnancy-related safety reports that were associated with the concomitant use of an anti-CGRP monoclonal antibody and a gepant, a combination treatment that still remains debated in the general population [17–19].

Our study has several limitations. Firstly, disproportionality analysis is a hypothesis-generating method that does not allow definitive conclusions to be drawn on drug safety. Secondly, the lack of exposure data and the unquantified under-reporting prevent from calculating the incidence of any drug toxicity. Lastly, due to the voluntary nature of spontaneous reporting, clinical details of VigiBase^®^ safety reports are not available, therefore it is not possible to know the exact time of drug exposure in pregnancy and the duration of treatment with CGRP-A during pregnancy.

## Conclusions

This study showed that, to date, there are no signals of increased spontaneous reporting with CGRP-A compared to triptans in relation to pregnancy in VigiBase^®^. Disproportionality analyses depend however on the progressively increasing number of safety reports gathered in the spontaneous reporting system. Therefore, future disproportionality analyses need to be performed and complemented with pregnancy pharmacovigilance studies on patient registries and other investigations on large-scale collaborative projects such as the Innovative Medicines Initiative (IMI) ConcePTION project [20].

### Supplementary Information

**Additional file 1: Supplementary Figure 1. **Selection of safety reports used as comparator group in disproportionality analyses.

**Additional file 2: Supplementary Figure 2. **Consort diagram showing the selection process of safety reports with CGRP antagonists included in the study cohort.

**Additional file 3: Supplementary Table 1. **Computation of reporting odds ratios and 95% confidence intervals in disproportionality analyses.

## Data Availability

The datasets used and analysed during the current study are available from the corresponding author on reasonable request.

## Abbreviations

CGRP: calcitonin gene-related peptide
CGRP–: A calcitonin gene-related peptide antagonists
CI: Confidence interval
IMI: Innovative Medicines Initiative
MedDRA: Medical dictionary for regulatory activities
ROR: Reporting odds ratio
SMQ: Standardized MedDRA query
