# Supplementary material for: Calcitonin gene-related peptide antagonists in pregnancy: a disproportionality analysis in VigiBase®
Source: J Headache Pain. 2024 Jan 19;25(1):10. doi: 10.1186/s10194-024-01715-4 (PMC10799383; doi:10.1186/s10194-024-01715-4)
Supplement: Supplementary file 1 — Additional file 1: Supplementary Figure 1. Selection of safety reports used as comparator group in disproportionality analyses. [file 10194_2024_1715_MOESM1_ESM.docx]

**Supplementary Figure** **1** Selection of safety reports used as comparator group in disproportionality analyses.


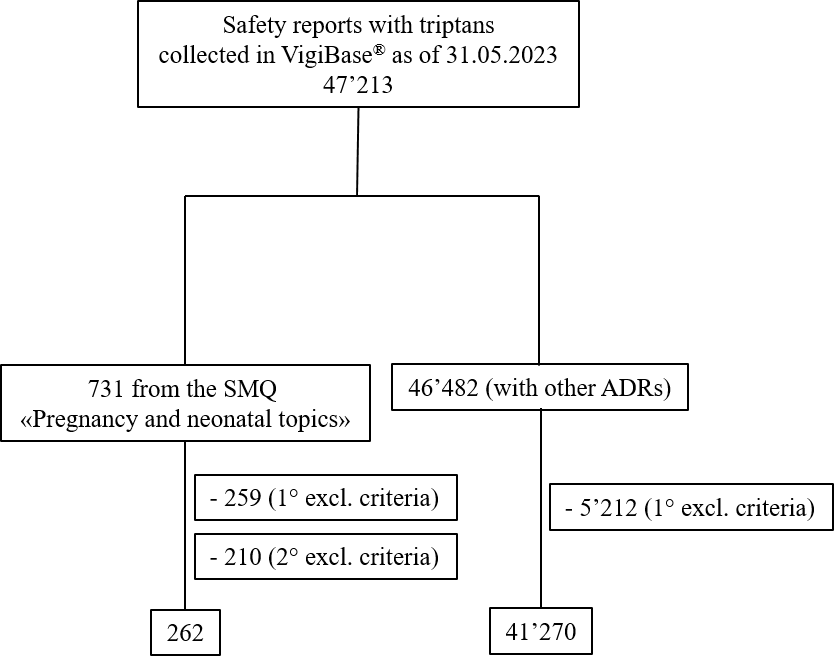


*Abbreviations:* SMQ, Standardised MedDRA Query; ADRs, adverse drug reactions
