# Supplementary material for: Calcitonin gene-related peptide antagonists in pregnancy: a disproportionality analysis in VigiBase®
Source: J Headache Pain. 2024 Jan 19;25(1):10. doi: 10.1186/s10194-024-01715-4 (PMC10799383; doi:10.1186/s10194-024-01715-4)
Supplement: Supplementary file 2 — Additional file 2: Supplementary Figure 2. Consort diagram showing the selection process of safety reports with CGRP antagonists included in the study cohort. [file 10194_2024_1715_MOESM2_ESM.docx]

**Supplementary Figure 2** Consort diagram showing the selection process of safety reports with CGRP antagonists included in the study cohort.


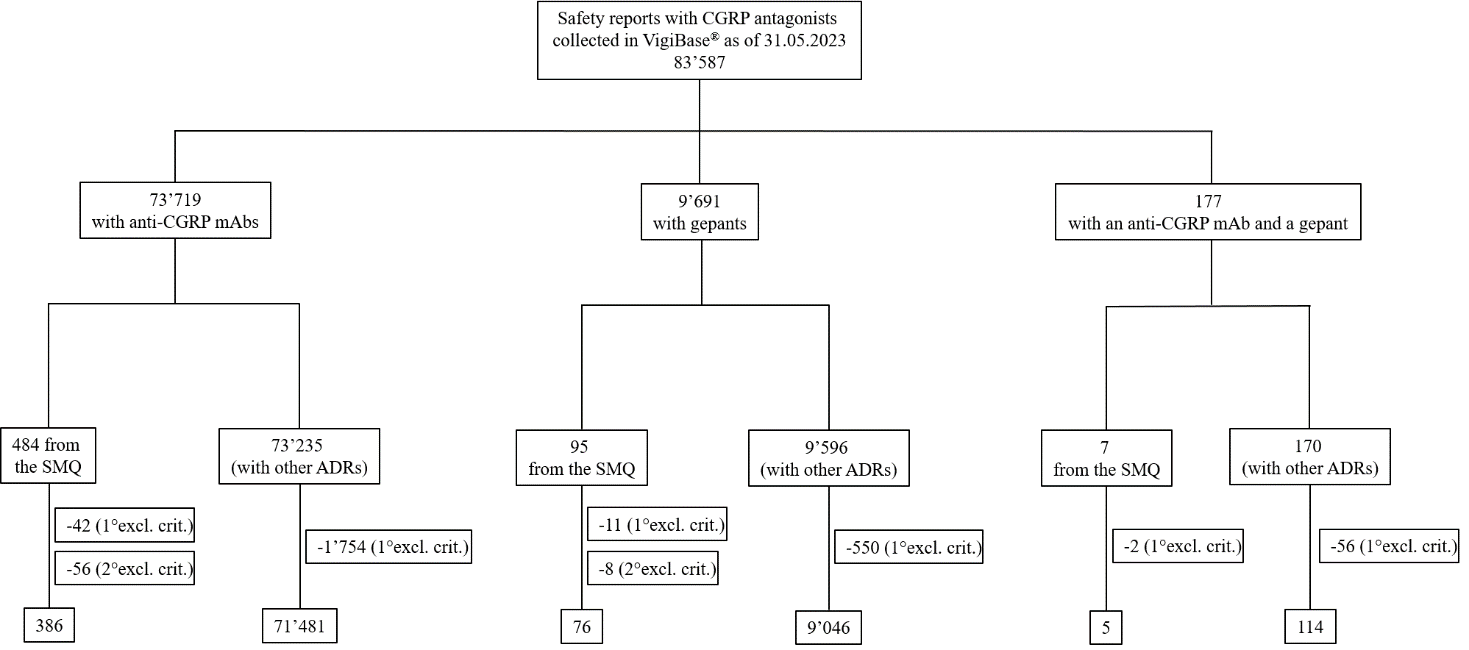


*Abbreviations:* CGRP, calcitonin gene-related peptide; mAbs, monoclonal antibodies; SMQ, Standardized MedDRA Query (“pregnancy and neonatal topics”); ADRs, adverse drug reactions; excl. crit., exclusion criterion (safety reports with additional suspected/interacting drugs beyond those of interest and safety reports lacking specific terms referring to exposure in pregnancy, respectively)
