# Supplementary material for: Calcitonin gene-related peptide antagonists in pregnancy: a disproportionality analysis in VigiBase®
Source: J Headache Pain. 2024 Jan 19;25(1):10. doi: 10.1186/s10194-024-01715-4 (PMC10799383; doi:10.1186/s10194-024-01715-4)
Supplement: Supplementary file 3 — Additional file 3: Supplementary Table 1. Computation of reporting odds ratios and 95% confidence intervals in disproportionality analyses. [file 10194_2024_1715_MOESM3_ESM.docx]

**Supplementary Table** **1** Computation of reporting odds ratios and 95% confidence intervals in disproportionality analyses.

| **Disproportionality analysis** | | **(a)** | **(b)** | **(c)** | **(d)** | **ROR [95% CI]** |
| --- | --- | --- | --- | --- | --- | --- |
| CGRP antagonists  vs triptans | All safety reports | 467 | 80641 | 262 | 41270 | 0.91 [0.78-1.06] |
|  | Safety reports reporting any pregnancy outcomes | 207 | 80641 | 195 | 41270 | 0.54 [0.45-0.66] |
|  | Safety reports reporting foetal/neonatal outcomes | 127 | 80641 | 123 | 41270 | 0.53 [0.41-0.68] |
| CGRP antagonists  vs triptans, since 2018 | All safety reports | 467 | 80641 | 91 | 9015 | 0.57 [0.46-0.72] |
|  | Safety reports reporting any pregnancy outcomes | 207 | 80641 | 64 | 9015 | 0.36 [0.27-0.48] |
|  | Safety reports reporting foetal/neonatal outcomes | 127 | 80641 | 35 | 9015 | 0.41 [0.28-0.59] |
| Anti-CGRP mAbs  vs triptans, since 2018 | All safety reports | 386 | 71481 | 91 | 9015 | 0.54 [0.43-0.67] |
|  | Safety reports reporting any pregnancy outcomes | 192 | 71481 | 64 | 9015 | 0.38 [0.29-0.50] |
|  | Safety reports reporting foetal/neonatal outcomes | 122 | 71481 | 35 | 9015 | 0.44 [0.30-0.64] |
| Gepants  vs triptans, since 2018 | All safety reports | 76 | 9046 | 91 | 9015 | 0.83 [0.61-1.13] |
|  | Safety reports reporting any pregnancy outcomes | 14 | 9046 | 64 | 9015 | 0.22 [0.12-0.39] |
|  | Safety reports reporting foetal/neonatal outcomes | 4 | 9046 | 35 | 9015 | NC |

(a) Number of safety reports of interest reported with CGRP antagonists

(b) Number of safety reports reporting events other than those of interest with CGRP antagonists

(c) Number of safety reports of interest reported with triptans

(d) Number of safety reports reporting events other than those of interest with triptans

*Abbreviations:* CGRP, calcitonin gene-related peptide; ROR, reporting odds ratio; CI, confidence interval; mAbs, monoclonal antibodies; NC, not computable (less than 5 safety reports)
